# Supplementary figures and images for: CRISPR Spacers Indicate Preferential Matching of Specific Virioplankton Genes
Source: mBio. 2019 Mar 5;10(2):e02651-18. doi: 10.1128/mBio.02651-18 (PMC6401485; doi:10.1128/mBio.02651-18)

A. Preliminary  
CRISPR Search

B. CRISPR  
Validation

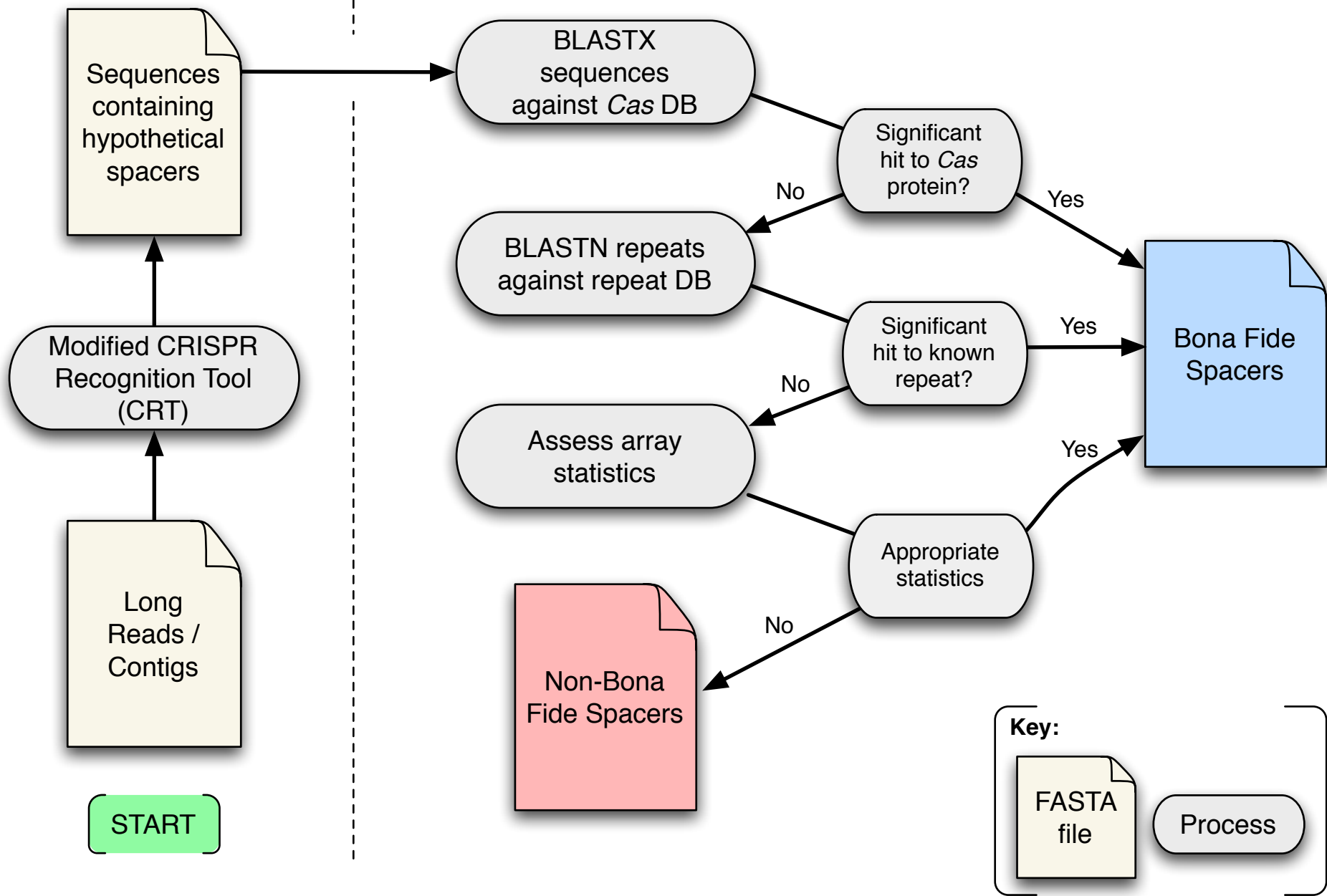

Supplement: FIG S1 [file mBio.02651-18-sf001.pdf]

**A**

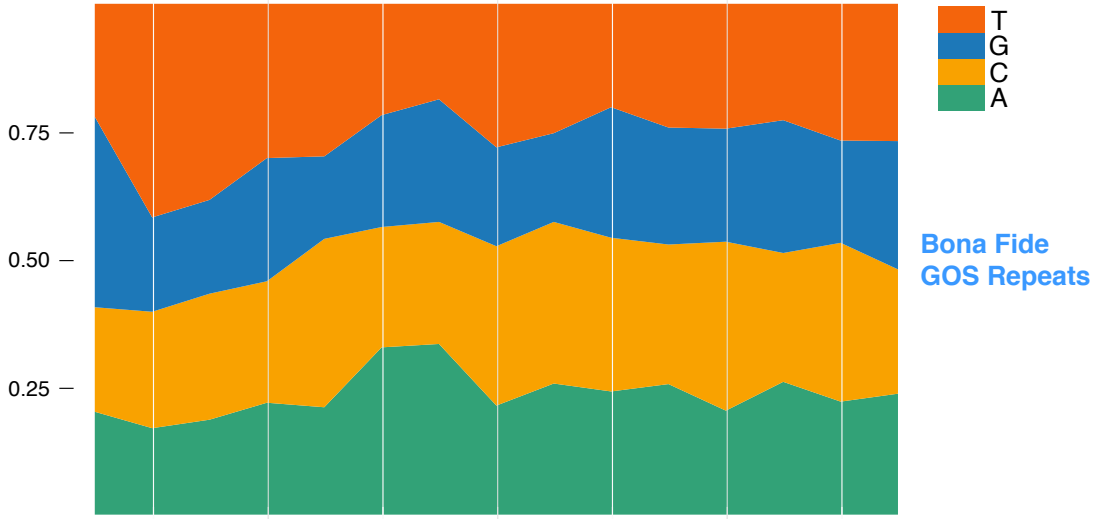

**B**

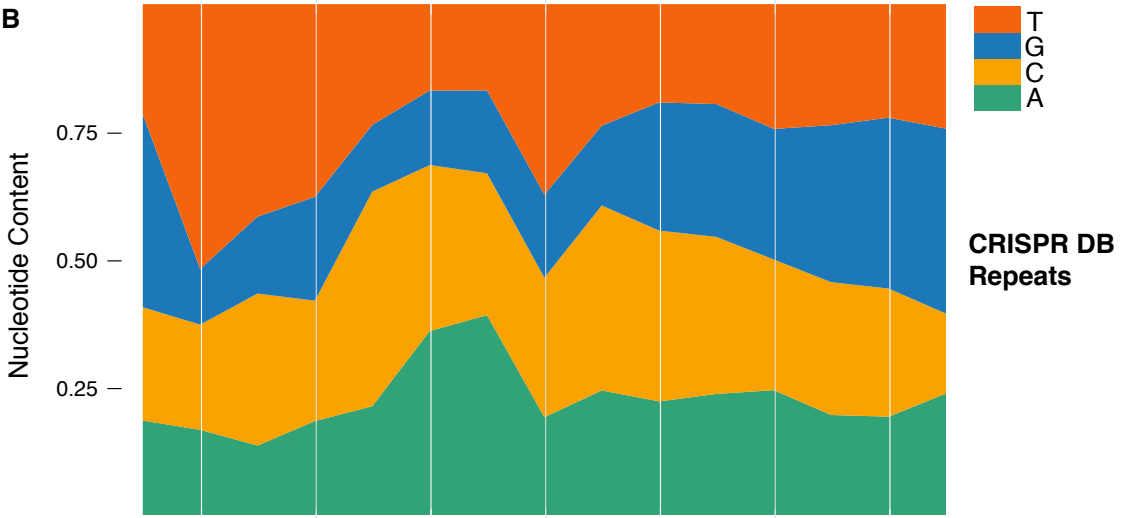

**C**

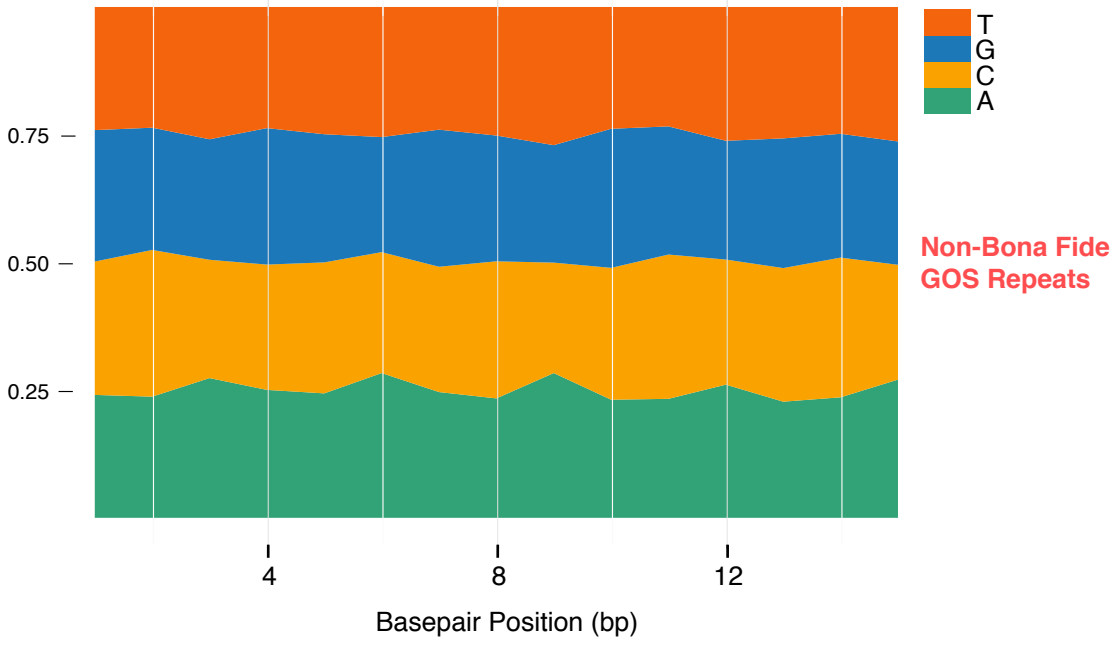

Supplement: FIG S2 [file mBio.02651-18-sf002.pdf]

A

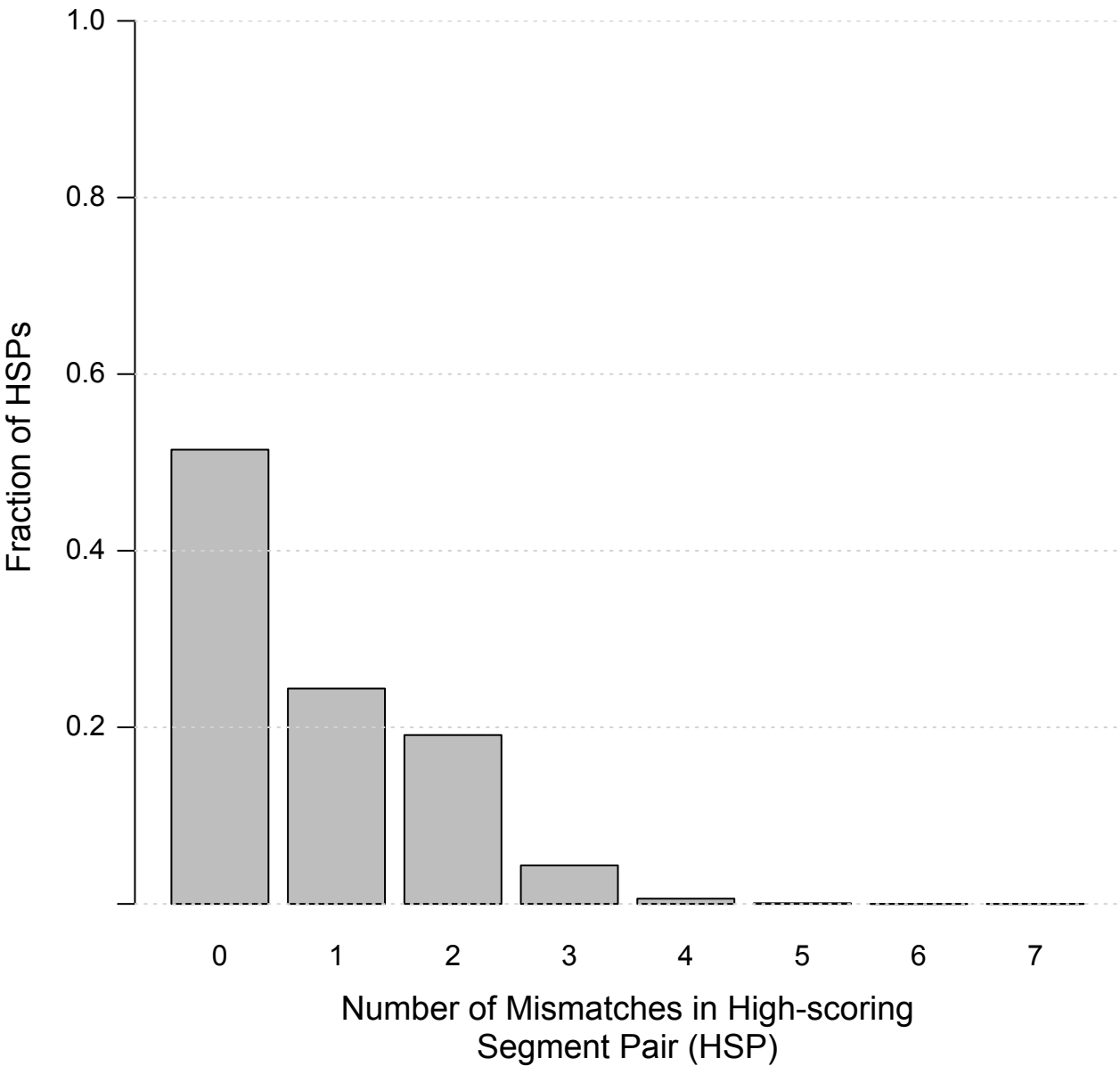

B

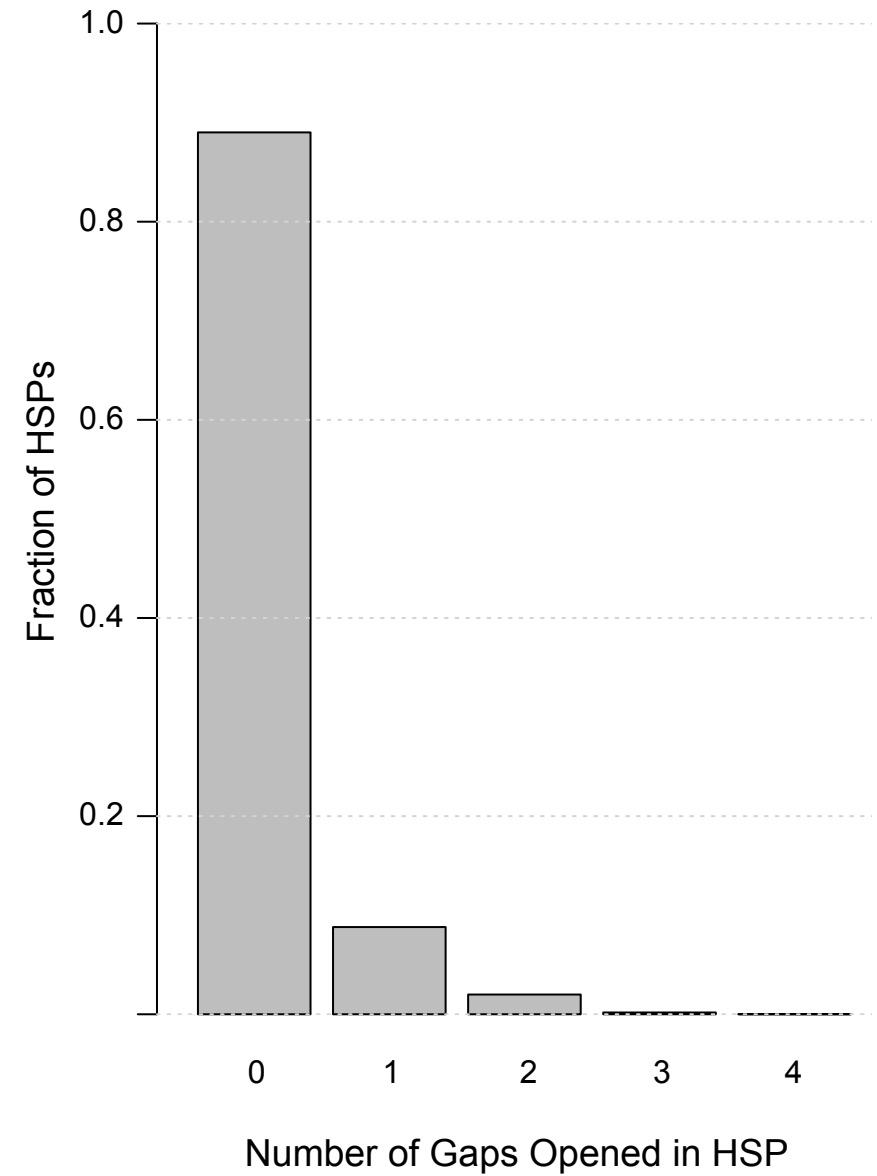

Supplement: FIG S3 [file mBio.02651-18-sf003.pdf]
