# Supplementary material for: CRISPR Spacers Indicate Preferential Matching of Specific Virioplankton Genes
Source: mBio. 2019 Mar 5;10(2):e02651-18. doi: 10.1128/mBio.02651-18 (PMC6401485; doi:10.1128/mBio.02651-18)
Supplement: TABLE S1 [file mBio.02651-18-st001.pdf]

Table S1. Bacterial genome sequences used in the construction of the mock metagenomes.

Organism	% GC	Genome Size (Mbp)	Spacers	Arrays
Escherichia coli str. K-12 substr. MG1655	51	4.6	18	2
Streptococcus salivarius JIM8777	40	2.2	32	1
Neisseria meningitidis 8013	51	2.3	25	1
Yersinia pestis A1122	48	4.5	16	3
Chlorobium tepidum TLS	57	2.1	62	2
Chlamydia trachomatis F/SW5	41	1.0	-	-
Ruegeria pomeroyi DSS-3	64	4.1	-	-
Bacillus thuringiensis HD-789	35	5.5	-	-
Bordetella pertussis CS	68	4.1	-	-
Acetobacter pasteurianus IFO 3283-01	53	2.9	-	-
