# Supplementary material for: CRISPR Spacers Indicate Preferential Matching of Specific Virioplankton Genes
Source: mBio. 2019 Mar 5;10(2):e02651-18. doi: 10.1128/mBio.02651-18 (PMC6401485; doi:10.1128/mBio.02651-18)
Supplement: TABLE S2 [file mBio.02651-18-st002.pdf]

Table S2. CRISPR finding tool performance Spacers found in the artificial 454 pyrosequencing metagenome using available CRISPR discovery tools.

Program	Spacers in Dataset	Spacers Detected	True Positives	False Positives	False Negatives	Sensitivity	Precision
CASC - Conservative	1930	981	802	179	1128	0.42	0.82
CASC - Liberal	1930	1623	1108	515	822	0.57	0.68
CRISPR Finder	1930	-	-	-	-	-	-
metaCRT	1930	2631	1225	1406	705	0.63	0.47
PILER-CR	1930	1483	1070	413	860	0.55	0.72
