# Supplementary material for: CRISPR Spacers Indicate Preferential Matching of Specific Virioplankton Genes
Source: mBio. 2019 Mar 5;10(2):e02651-18. doi: 10.1128/mBio.02651-18 (PMC6401485; doi:10.1128/mBio.02651-18)
Supplement: TABLE S3 [file mBio.02651-18-st003.pdf]

Table S3. Spacers found in the artificial Illumina metagenome using available CRISPR discovery tools.

Program	Spacers in Dataset	Spacers Detected	Spacer Coverage in Dataset	Spacer Coverage Detected	True Positives	False Positives	False Negatives	Sensitivity	Precision
CASC - Conservative	153	153	42,349	41,095	153	0	0	1.00	1.00
CASC - Liberal	153	153	42,349	41,095	153	0	0	1.00	1.00
CRISPR Finder	153	216	42,349	62,418	153	63	0	1.00	0.71
metaCRT	153	365	42,349	96,503	153	212	0	1.00	0.42
PILER-CR	153	146	42,349	39,222	138	8	15	0.90	0.95
